# Automatic registration of multi-modal microscopy images for integrative analysis of prostate tissue sections

**DOI:** 10.1186/1471-2407-13-408

**Published:** 2013-09-05

**Authors:** Giuseppe Lippolis, Anders Edsjö, Leszek Helczynski, Anders Bjartell, Niels Chr Overgaard

**Affiliations:** 1 Department of Clinical Sciences, Division of Urological Cancers, Skåne University Hospital, Lund University, Malmö, Sweden; 2 University and Regional Laboratories Region Skåne, Clinical Pathology, Malmö, Sweden; 3 Centre for Mathematical Sciences, Lund University, Lund, Sweden

**Keywords:** Multiplex analysis, Histological sections, Hematoxylin & Eosin, p63/AMACR, Time resolved fluorescence imaging, Image registration, Scale invariant feature transform, Prostate cancer

## Abstract

**Background:**

Prostate cancer is one of the leading causes of cancer related deaths. For diagnosis, predicting the outcome of the disease, and for assessing potential new biomarkers, pathologists and researchers routinely analyze histological samples. Morphological and molecular information may be integrated by aligning microscopic histological images in a multiplex fashion. This process is usually time-consuming and results in intra- and inter-user variability. The aim of this study is to investigate the feasibility of using modern image analysis methods for automated alignment of microscopic images from differently stained adjacent paraffin sections from prostatic tissue specimens.

**Methods:**

Tissue samples, obtained from biopsy or radical prostatectomy, were sectioned and stained with either hematoxylin & eosin (H&E), immunohistochemistry for p63 and AMACR or Time Resolved Fluorescence (TRF) for androgen receptor (AR).

Image pairs were aligned allowing for translation, rotation and scaling. The registration was performed automatically by first detecting landmarks in both images, using the scale invariant image transform (SIFT), followed by the well-known RANSAC protocol for finding point correspondences and finally aligned by Procrustes fit. The Registration results were evaluated using both visual and quantitative criteria as defined in the text.

**Results:**

Three experiments were carried out. First, images of consecutive tissue sections stained with H&E and p63/AMACR were successfully aligned in 85 of 88 cases (96.6%). The failures occurred in 3 out of 13 cores with highly aggressive cancer (Gleason score ≥ 8). Second, TRF and H&E image pairs were aligned correctly in 103 out of 106 cases (97%).

The third experiment considered the alignment of image pairs with the same staining (H&E) coming from a stack of 4 sections. The success rate for alignment dropped from 93.8% in adjacent sections to 22% for sections furthest away.

**Conclusions:**

The proposed method is both reliable and fast and therefore well suited for automatic segmentation and analysis of specific areas of interest, combining morphological information with protein expression data from three consecutive tissue sections. Finally, the performance of the algorithm seems to be largely unaffected by the Gleason grade of the prostate tissue samples examined, at least up to Gleason score 7.

## Background

Prostate cancer (PCa) is the second most common cancer in men worldwide. About 910.000 new cases were recorded in 2008 accompanied with 258.000 deaths. According to current estimates, the incidence of PCa is expected to double by 2030 [1].

Analysis of the microscopic features of the prostate is vital for clinical management of PCa patients, both with respect to diagnosis and prognosis. Today, PCa is commonly diagnosed by a uropathologist carefully examining at least ten transrectal ultrasonography (TRUS)-guided prostate biopsies using conventional brightfield microscopy [2]. Manual morphological analysis is also carried out on whole-mount tissue sections after radical prostatectomy (RP), which may provide valuable prognostic information about outcome of the disease. The most important assessment of the morphology is to determine tumor grade according to the Gleason system [3]. Moreover, considerable research efforts have been directed towards the analysis of tissue sections for assessing the presence of proteins (biomarkers) which can potentially be related to the development and progression of the disease [4]. The study of tissue biomarkers has been expanding since the implementation of Tissue Micro Arrays (TMAs) [5]. Such arrays can contain several hundreds of tissue samples (cores) and have paved the way for high-throughput studies of predictive tissue biomarkers [6].

A common research objective is to investigate the expression of several biomarkers on a stack of consecutive tissue sections. Moreover it is important to be able to recognize specific tissue compartments (benign vs. cancer, epithelial vs. stromal cells, cell cytoplasm vs. nuclei) where such biomarkers are expressed, as this might be related to different states of the disease. There is an unmet need to combine morphological information with protein expression analysis coming from consecutive tissue sections. An automated approach would make this procedure fast and suitable for the study of multiple features on large TMAs.

The aim of our paper is to investigate the feasibility for an integrative analysis through automated registration of digital images of consecutive histological prostate sections stained and visualized with different modalities.

Manual evaluation of histological sections is time-consuming and highly dependent on the user’s experience, resulting in high inter- and intra-variability [7]. However the improvement in technology and the access to larger storing facilities in the last decade have led to the creation of digital slide scanners and large digital archives [8]. This paves the way for the use of Image Analysis techniques to handle histological images.

Automated registration of histological sections (stained with the same modality) has been attempted on cervical carcinoma by Braumann et al. [9], while automated registration of multimodal microscopy with application to PCa is considered in a recent paper by Kwak et al. [10]. Their aim was to register pairs of images, from light microscopy and infrared spectroscopy, in order to extract morphological features for use in the classification of cancer versus non-cancer cases. The registration is intensity based, leading to a minimization of a non-convex similarity measure over a four-dimensional space of transformation parameters. This problem is solved using the Nelder-Meade simplex method, which is a local search technique. In contrast, our registration method is landmark-based, with the landmarks coming from Scale Invariant Feature Transform (SIFT), which has the advantage of speed. Moreover, landmark-based methods look for similar features in the image pair rather than dissimilarities and may therefore succeed even in the presence of noise and occlusions. SIFT works with gray-scale images, therefore using more of the original image information when compared to Kwak et al. [10], where only binary (black-white) images were used.

A number of papers explore the possibility to integrate information from *in vivo* imaging (ex. PET, MRI) with histology [11], and analysis of sequential immunofluorescence staining for assessing several biomarkers [12].

Multiple studies apply SIFT [13] for landmark-based registration of medical images. The earliest of such studies was performed by Chen et al. [14], where unimodal registration was considered. Their experiments are of a very preliminary nature. Other applications are found in Tang et al. [15] and Wei et al. [16]. The former consider alignment of stem cell images whereas the latter is concerned with registration of retinal images, which differs from our problem in that it requires registration transformations of another type (quadric transformations). Another relevant contribution is described by Zhan et al. [17] where texture landmarks, found using scale-space methods, are used in the non-rigid registration (with thin plate splines) of prostate image pairs from histological and MR specimens. For a pair of images, the determination of landmark correspondences and the best registration transformation is found simultaneously by solving a non-linear optimization problem in a large number of variables. Evaluation was carried out for five image pairs.

**Figure 1:**
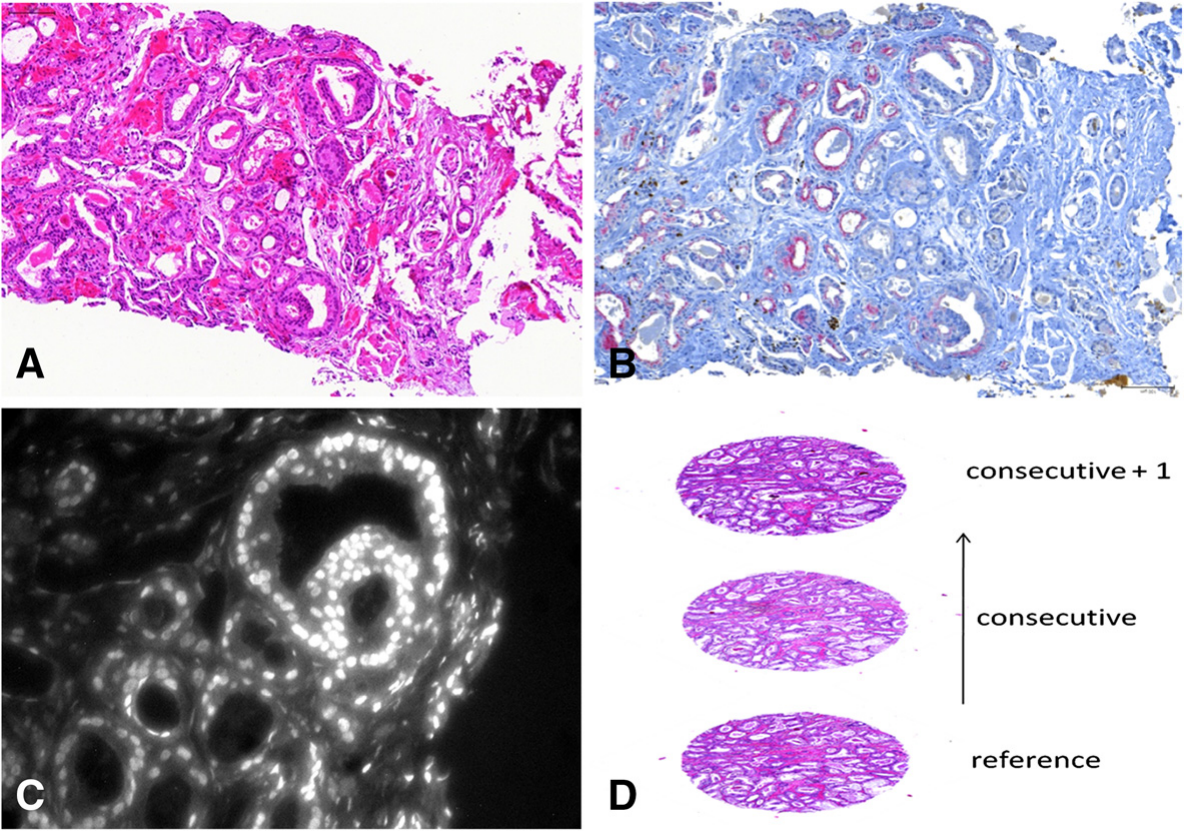
**Tissue sections and staining techniques. A**, H&E. Nuclei stained in blue (Hematoxylin); Eosin stains all other structures in various shades of pink. This staining shows the morphological features of the tissue and is used by uropathologists to diagnose cancer and grade its aggressiveness (Gleason score). **B**, p63/AMACR. p63 is a protein present in the basal cells of benign glands and appears brown while AMACR protein is present in the cytoplasm of cancer cells and appears red. This staining is used to confirm the diagnosis when H&E is not clear. **C**, TRF for AR. AR is present in cell nuclei and its expression may be related to the status of the disease. AR was detected through TRF, which allows for quantification of the fluorescence signal. Modalities in **A**, **B**, **C** are used in Experiment 1 and 2. **D**, schematics of a stack of consecutive tissue sections stained with H&E, such as the one used in experiment 3. The images size is typically 1000x1000 pixels.

The focus of the present paper is the alignment problem for triplets of images produced with different modalities. In particular we have used two pairs of images. One pair includes two images from consecutive sections stained respectively for hematoxylin and eosin (H&E) and antibodies directed against p63 and Alpha-methylacyl-CoA racemase (AMACR), a combination of proteins used in routine clinical diagnostics to identify basal cells and high grade prostate intraepithelial neoplasia (HGPIN)/PCa cells, respectively. Importantly, these 2 stainings give morphological information and a possibility to identify cancer areas. The other pair includes one H&E image and one Time Resolved Fluorescence (TRF) for Androgen Receptor (AR) obtained from the same section after washing off the H&E staining. This gives information about the status of a potential biomarker (AR) within the prostate. All these modalities are presented in Figure 1. We use SIFT-landmarks, RANSAC and Procrustes alignment, which yields an equally reliable yet faster method for registration than that which has previously been described in [10]. In our work, we have used images coming from real patient material collected and processed at our institution. The staining techniques were optimized in order to generate strong and specific detectable signals with minimal background noise.

## Methods

### Tissue acquisition and processing

Tissue samples came from two sources: RP for curative purpose and needle biopsies taken for diagnostic purposes. From the prostatectomy material cores with 1 mm diameter were punched out of relevant blocks and organized in a TMA format. Core needle biopsies are up to 15 mm long and 1 mm wide tissue samples. After the acquisition procedure both types of material were fixed in formalin and embedded in paraffin.

To conduct the study, 4 μm sections were cut from the paraffin blocks and mounted on slides. The pre-processing before staining includes deparaffinization through xylene and ethanol with decreasing concentration, followed by rehydration and antigen retrieval to allow the antibodies to bind to the proteins of interest. The process described above has been performed manually and the accuracy of each step can affect the quality of the final results and introduce artifacts. For example, tissue samples can undergo mechanical deformation during handling and an incorrect preprocessing can cause poor staining and therefore inferior images.

The procedure was done strictly in compliance with the Helsinki Declaration after approval from the Regional Ethical Review Board at Lund University.

### Staining

In Experiment 1, a TMA containing 88 cores was produced and sectioned. One section was stained for H&E followed by immunohistochemistry for p63/AMACR on the consecutive section. The H&E is a traditional and standardized method in which cellular nuclei are stained with a bluish shade while the cytoplasm is stained with different shades of pink. Slides stained with this procedure are generally used to determine the presence of cancer and assess its aggressiveness. The p63/AMACR is a double staining procedure in which the single basal cell layer surrounding a benign gland has a brown nuclear staining (p63), the cytoplasm in the majority of the cancer cells is stained with reddish shade (AMACR) and the rest of the tissue has different shades of blue. This staining helps the pathologist to spot the presence of cancer or pre-malignant lesions with HGPIN when the histological pattern is inconclusive.

For Experiment 2, sections from biopsies were stained with mouse monoclaonal anti-AR antibody (AR411) which was previously labelled with Europium for TRF. TRF is an evolution of conventional immuno-fluorescence. It uses lanthanide chelates (europium, terbium, etc.) as fluorophores [18]. The long decay times of these isotopes together with a gated acquisition system allow for the detection of a specific signal by excluding the autofluorescence phenomenon, thus obtaining a more linear quantification of the biomarker. Here, TRF is used for the quantification of tissue protein expression in specific compartments as previously shown [19]. After acquisition of images by TRF the AR411 antibody was washed off and the samples were further processed with H&E staining.

Finally, in Experiment 3, one TMA was built containing 50 cores from prostatectomies; four sections were cut, mounted on slides and stained for H&E. This TMA was used to validate results in Experiments 1 and 2 and to study the inner morphological variability of prostatic tissue.

### Gleason grading

A normal prostate is organized in glandular structures formed by a layer of basal cells and a layer of epithelial cells surrounding an empty space known as the lumen. Such glands are surrounded by connective tissue called stroma. In presence of cancer, this normal glandular structure is disrupted. The Gleason scoring was introduced in the 1960’s and updated in 2005 [20]. It is a system based on histological growth patterns of cancer cells. The Gleason grades (ranging from 1–5) of cancer cells from areas of two distinct growth patterns (two most prevalent) are summed up to form a Gleason score ranging from 2 to 10. A high Gleason grade, and thus Gleason score, is found in less differentiated tumours, that generally are more aggressive and have a poor prognosis [21].

In order to assess the ability of the algorithm to register a large range of images with various morphological characteristics, a pathologist evaluated H&E staining and assigned a Gleason score to each core.

### Image acquisition

The Mirax Scan (Carl Zeiss) equipped with Plan-Apochromat 20x/0.75 objective was used to take pictures of H&E and p63/AMACR stained sections.

For Experiment 1, we collected twenty times magnified (20x) images for each core resulting in a total of 88 image pairs (H&E and p63/AMACR in consecutive sections).

For Experiment 2, 106 images pairs (H&E and TRF) were collected. The Nikon Eclipse 600 equipped with an appropriate laser and programmed electronics (Signifer 1432 MicroImager; Perkin-Elmer Life Sciences; Wallac Oy) was used for TRF acquisition. In order to acquire the Europium signal, a filter with excitation and emission bands centered in 340 nm and 615 nm was used. TRF produced forty times maginified (40x) images.

For Experiment 3, we collected 20x images for each core of the four sections.

### Image registration

As described in Zitova et al. [22], our registration algorithm pipeline consists of four steps: (1) feature detection and extraction, (2) feature matching, (3) transformation function fitting and (4) image transformation and image resampling.

We first explain the steps (3) and (4), to fix terminology, and then move to SIFT (1) and RANSAC (2).

In our description a gray scale image *I* is a real valued function *I*:Ω → [0,1] defined in a planar region Ω, called the image domain, and whose value at a particular point (pixel) ***x****=* (*x*_1_, *x*_2_) is the gray level *I* (***x***).

Suppose now that we are given two images *I*_1_:Ω_1_ → [0,1] and *I*_2_:Ω_2_ → [0,1] where *I*_2_ depicts a scene which is similar to the one obtained if the scene in *I*_1_ is subjected to a similarity transformation, i.e., a mapping ***y****= T* (***x***) of the following form

$$T\left(x\right)=\left[\begin{array}{cc} a & -b\\ b & a \end{array}\right]\;\left(\begin{array}{c} x_{1}\\ x_{2} \end{array}\right)+\left(\begin{array}{c} t_{1}\\ t_{2} \end{array}\right).$$

Thus *T* is the combination of a scaling by the factor $\sqrt{a^{2}+b^{2}}$, a rotation by the angle arctan(*b/a*) and a translation by (*t*_1_*,t*_2_). We define the transformed image T*I_2_ : *Ω* → [0, 1] as the *pullback* of *I*_2_ by *T*, that is, by the formula T*I_2_(***x***) = *I*_2_(*T*(***x***)) if *T*(***x***) ∈ Ω_2_, otherwise T*I_2_(***x***) = 0. The objective is to find a map *T* such that T*I_2_(***x***) becomes as similar to *I*_1_ as possible. We do this by finding corresponding keypoints in the two images and then estimate the optimal mapping using Procrustes analysis.

Assume that we have found *N* point pairs ${\left\{\left(x^{i},y^{i}\right)\right\}}_{i=1}^{N}$ in the two images, such that ***y***^*i*^ ∈ Ω_2_ corresponds to ***x***^*i*^ ∈ Ω_1_ up to a small error ϵ^*i*^ after transformation:

$$y^{i}=T\left(x^{i}\right)+\epsilon^{i}\;\left(i=1,\ldots ,N\right),$$

where *T* is a similarity transformation of the above type. The desired mapping is the one which minimizes the sum of the squares of the errors: minT12∑i=1N ϵi2. Observe that if the transformation parameters are collected in a vector ***z*** = (*a*, *b*, *t*_1_, *t*_2_) then we may write, *T*(***x***) = *B*(***x***) ***z*** where *B*(***x***) is the matrix

$$B\left(x\right)=\left[\begin{array}{cc} x_{1} & -x_{2}\\ x_{2} & x_{1} \end{array}\;\begin{array}{cc} 1 & 0\\ 0 & 1 \end{array}\;\right]$$

The error becomes **ϵ**^*i*^ = ***y***^*i*^ − *B*(***x***^*i*^) ***z***, which is linear in ***z***. (This is possible only in two dimensions).

If we stack the **y-**vectors as *Y*^*T*^ = [(***y***^1^)^*T*^, …, (***y***^*N*^)^*T*^] and introduce the matrix *B* by *B*^*T*^ = [*B*(***x***^1^)^*T*^, …, *B*(***x***^*N*^)^*T*^] then one can see that the error-minimization becomes a classical least squares problem with respect to ***z***,

$$\min_{z}\frac{1}{2}{||Y-Bz||}^{2}$$

where ∣∣ ⋅ ∣∣ now denotes the norm in ***R***^2*xN*^. The desired mapping corresponds to the optimal ***z***, which is the solution the normal equations *B*^*T*^*B****z*** = *B*^*T*^*Y*. For this problem to be solvable we need at least two corresponding point pairs. This is sufficient if the pairs are nondegenerate, however, we use at least four point correspondences to get a more well-conditioned problem.

The corresponding keypoint pairs, used in the Procrustes alignment are found using SIFT and RANSAC in a classical manner, described briefly below.

SIFT [23] works by the following principle: first, keypoints are detected in the image. They are local extrema in space and scale when the image is embedded in its scale-space, and they have the property that they are stable under changes in illumination and view-point. Secondly, each such keypoint has a descriptor associated with it, as similar to a fingerprint. In this paper, the keypoint together with its descriptor is called a landmark. The descriptor consists of a 128-dimensional vector containing gradient statistics from eight directions in a 4 × 4 neighbourhood of the keypoint.

A Preliminary matching is then performed; assume we have found keypoints ***x***^*i*^, *i* = 1, …, *N*_1_, in *I*_1_ and ***y***^*j*^, *j* = 1, …, *N*_2_ in *I*_2_, together with their descriptors. Let *D* = [*d*_*ij*_] denote the *N*_1_ × *N*_2_ distance matrix, where *d*_*ij*_ denotes the Euclidean distance between the descriptors of ***x***^*i*^ and ***y***^*j*^ For each index *i*, the points ***x***^*i*^ and $y^{j^{*}}$, where $j^{*}=\underset{j}{\arg\;\min}\;d_{\mathrm{ij}}$, is called a *preliminary matching* if the following condition holds

$$\frac{\min_{j}d_{\mathrm{ij}}}{\min_{j\neq j*}d_{\mathrm{ij}}}<0.77.$$

This condition is known as Lowe’s criterion. It states that the nearest neighbor of the descriptor of ***x***^*i*^ in the set of descriptors of all the keypoints ***y***^*j*^ should be much closer than the next-nearest neighbor in order for the keypoint ***x***^*i*^ to be matched with $y^{j^{*}}$. We have applied the implementation of SIFT by Vedaldi and Fulkerson [24].

The set of preliminary matches found above may contain a significant percentage of false matches, usually referred to as outliers. The RANSAC algorithm invented by Fischler and Bolles [25] can be used to select a large subset of matches, called inliers, from the set of preliminary matches which is consistent with the registration model. RANSAC is a statistical approach where a small number of preliminary matches are selected at random from the set of preliminary matches and used to estimate a model; in our case we use four preliminary matches to estimate a Procrustes alignment. Using this alignment transformation all keypoints in the first image are transformed into the second image. If a transformed keypoint is within 5 pixels of the keypoint to which it has been matched in the preliminary matching, then the preliminary matching of this pair of keypoints is considered to be an inlier. The number of such inliers is then recorded. This procedure is repeated (in our case 100 repetitions) and the model is chosen which has the highest number of inliers. The final alignment is then estimated by Procrustes analysis using all of the matches in the set of best inliers.

### The evaluation procedure

**Figure 2:**
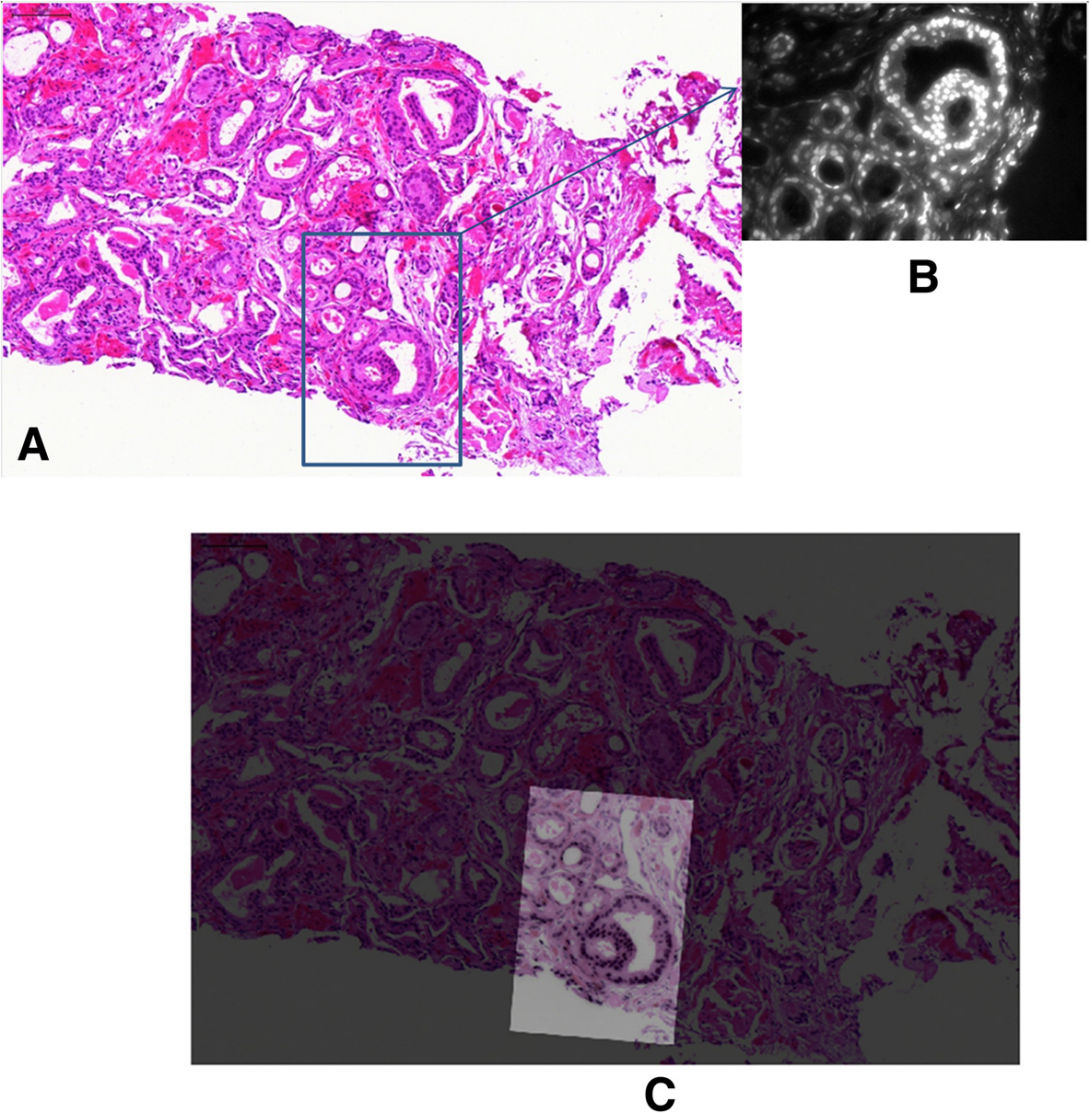
**Successful alignment of H&E and AR. A**, tissue section of a prostate biopsy, stained for H&E (20x magnification); **B**, the same tissue section stained for AR using TRF (40x). **C**, Successful alignment shown as an overlay of image **B** onto image **A**. The staining procedure was the following: first the tissue section was stained for AR and pictures acquired through TRF, then the AR was washed off, the section was stained for H&E and a new picture was acquired through brightfield microscopy. Considering that AR is the protein to be quantified, it is important that AR expression is preserved and therefore that the tissue is minimally stressed. Since the tissue is processed twice and this might alter its structure and protein content, we have performed AR as the first staining. H&E on the other hand did not seem to be highly influenced by intermediate steps.

The proposed registration method has been tested in three different experiments, each addressing different image alignment problems. In all three experiments the quality of the registration was evaluated visually. A registration was defined as *correct* if the computed transformation was able to overlay the two images in such a way that corresponding areas of interest were visually confirmed to line up appropriately. An example of an image overlay is shown Figure 2. Each visual evaluation was performed by two independent authors.

Visual evaluation has the obvious drawback of being subjective, however was chosen in order to save time. Since the human eye is very good at detecting visual inconsistencies we believe that visual evaluation is an appropriate method for evaluating many registrations within a limited amount of time.

We do not, however, rely entirely on visual inspection. In the first of our three experiments we have also performed an extensive quantitative evaluation of the results. Note that the first experiment contains potentially the most challenging of the three registration problems considered in this work since the image pairs consist of adjacent tissue sections stained with different modalities. The quantitative evaluation has two purposes, first of which is to measure the quality of the automatic registration results. Second, the quantitative evaluation was used to show the reliability of the visual evaluation, which was employed in experiments 2 and 3.

With regards to the procedure of the quantitative evaluation, in the 85 of the 88 cases where visual evaluation has classified the automatic registration as correct, the resulting registration transformation is compared to the transformation obtained from Procrustes analysis using manually detected keypoint pairs. More specifically, for each image pair, multiple keypoint pairs were found manually. If the images contained prominent salient features, three to four keypoints were used, otherwise five keypoints were chosen. Procrustes analysis was then performed and the corresponding transformation *T*_*manual*_ was recorded.

Next, the intrinsic uncertainty of the manual registration is estimated. The intrinsic uncertainty is a positive number *ϵ*_*manual*_ defined in the following way: let {***x***^*i*^, ***y***^*i*^}, *i* = 1, …, *N*, denote the *N* manually detected pairs of corresponding keypoints and define the residuals **ϵ**^***i***^ = ***y***^*i*^ − *T*_*manual*_(***x***^*i*^). The residuals have mean value of zero,

1N∑i=1N ϵi=0,

by the construction of *T*_*manual*_. The intrinsic uncertainty in the manual registration defined as the standard deviation *ϵ*_*manual*_ of the lengths of the residuals, i.e.,

ϵmanual2=1N−1∑i=1N ϵi2.

Note that this is, up to a fixed multiple, the quantity that is minimized in the Procrustes analysis in order to determine the optimal transformation *T* = *T*_*manual*_.

The next step is to use the proposed automatic registration method to compute the alignment transformation *T*_*auto*_. In order to estimate the uncertainty in the automatic registration we use the manually detected keypoints {***x***^***i***^, ***y*** ^***i***^} once more to compute the residuals $\epsilon_{\mathrm{auto}}^{i}=y^{i}-T_{\mathrm{auto}}\left(x^{i}\right)$. We then define the uncertainty *ϵ*_*auto*_ as the positive number given by

ϵauto2=1N−1∑i=1N ϵautoi2.

This is the same expression used in the definition of the intrinsic uncertainty of the manual registration, except that this time the automatically determined transformation *T*_*auto*_ is used to map the manually detected keypoints {***x***^*i*^} from the first image into the second image. Note that, since the transformation *T*_*manual*_ is defined as the similarity transformation which minimizes the expression ϵ2=1N−1∑i=1Nϵ i2 then the inequality *ϵ*_*manual*_ ≤ *ϵ*_*auto*_ is always satisfied.

We define an automatic registration as quantitatively correct if the following condition is satisfied,

$$\epsilon_{\mathrm{auto}}\leq \epsilon_{\mathrm{manual}}+5\;\mathrm{pixels}$$

The tolerance of five pixels corresponds to the tolerance used in the RANSAC sub-procedure of the automatic method. It should also be noted that the average size of a cell nucleus in the images used in our experiments was approximately 5 pixels. This criterion is used to evaluate the performance of the automatic registration method in experiment 1. If the number of quantitatively correct registrations is a large percentage of the images in the sample, then we will conclude that automatic registration is as good as manual registration. Moreover, if the number of quantitatively correct registrations is found to be almost the same as the number of visually correct registrations, then we will conclude that visual evaluation is reliable for our purpose in all three experiments.

## Results

### Experiment 1

**Figure 3:**
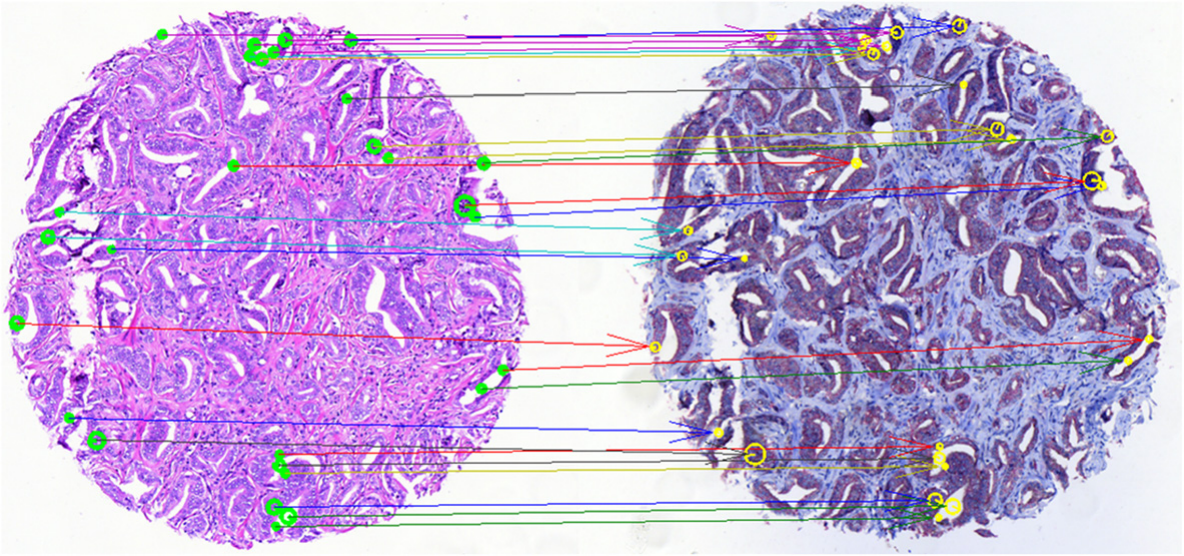
**Successful alignment of H&E (left) and p63/AMACR (right).** Initial keypoints ≈ 1000 in each image, preliminary matches = 34, best inliers = 31. The arrows link the matching inliers on the two images after rotation and scaling of the right image. In a perfect alignment the arrows would be parallel. This, however, is unrealistic in practice.

In Experiment 1, 85 out of 88 images (96.6%) were correctly aligned according to visual evaluation (Figure 3).

Table 1 shows the average number of keypoints, initial matches, best inliers and success rate.

**Table 1:** Experiments 1

	**H&E kyp**	**p63/AMACR kyp**	**Initial matches**	**(Best inliers)/initial matches**	**Success rate (#correct/#tot)**
Exp 1	953	1327	67	66%	96.6% (85/88)

Keypoints (kyp), initial matches, best inliers and success rate in experiment 1.

We also analyzed the location of the matching keypoints and found out that 32.6% of them are present within the lumina, 19.4% in the glandular epithelial layer and 48% in mixed areas (between glands).

An independent observer evaluated the H&E sections and assigned a Gleason score to each core. The algorithm correctly aligns 10/10 cores containing stroma, 37/37 containing benign tissue, 14/14 containing tumors with Gleason score 6, 14/14 containing Gleason score 7 tumors (eight cores with Gleason score 3+4 and six containing Gleason score 4+3), 10/13 containing tumors with a Gleason score higher than 7 (Table 2).

**Table 2:** Experiment 1; performance of the algorithm in different histological classes

	**Stroma**	**benign**	**Gleason 6**	**Gleason 7**	**Gleason >7**
Correct/total	10/10	37/37	14/14	14/14	10/13

The results suggest that Gleason score might be influential for the algorithm success only for very aggressive cases.

A qualitative evaluation of the 85 images that were classified as correctly aligned by visual evaluation was performed. The automatic and manual registration methods were compared for each image pair by computing the uncertainties * ϵ*_*auto*_ and * ϵ*_*maual*_, defined in the Methods Section. Recall that we define a registration as being quantitatively correct if

$$\epsilon_{\mathrm{auto}}\leq \epsilon_{\mathrm{manual}}+\mathrm{tol},$$

where the tolerance *tol* = *5* pixels was used. Using this criterion we found that 82 of the 88 (93.2%) of the image pairs are correctly aligned. Thus, three of the image pairs which were originally considered correctly aligned by the visual evaluation were rejected by the quantitative evaluation. It should be noted that two of these image pairs failed to satisfy the quantitative criterion by as narrow a margin as one fifth of a pixel or less. For comparison, the quantitative criterion was employed with *tol* = 4 pixels, which gave 80 of 88 (90.9%) correct alignments, and with *tol* = 6 pixels, which resulted in 84 of 88 (95.5%) correct alignments.

We also computed the statistics of the intrinsic uncertainty of the manual registration and found the mean value *μ*(*ϵ*_*manual*_) = 3.38 pixels and standard deviation of *σ*(*ϵ*_*manual*_) = 2.60 pixels, hence the estimate *ϵ*_*manual*_ = 3.4 ± 2.6 pixels. The corresponding statistics for the automatic registration is *ϵ*_*auto*_ = 5.0 ± 3.3 pixels. These estimates should be set in relation to our chosen tolerance *tol* = 5 pixels.

### Experiment 2

**Figure 4:**
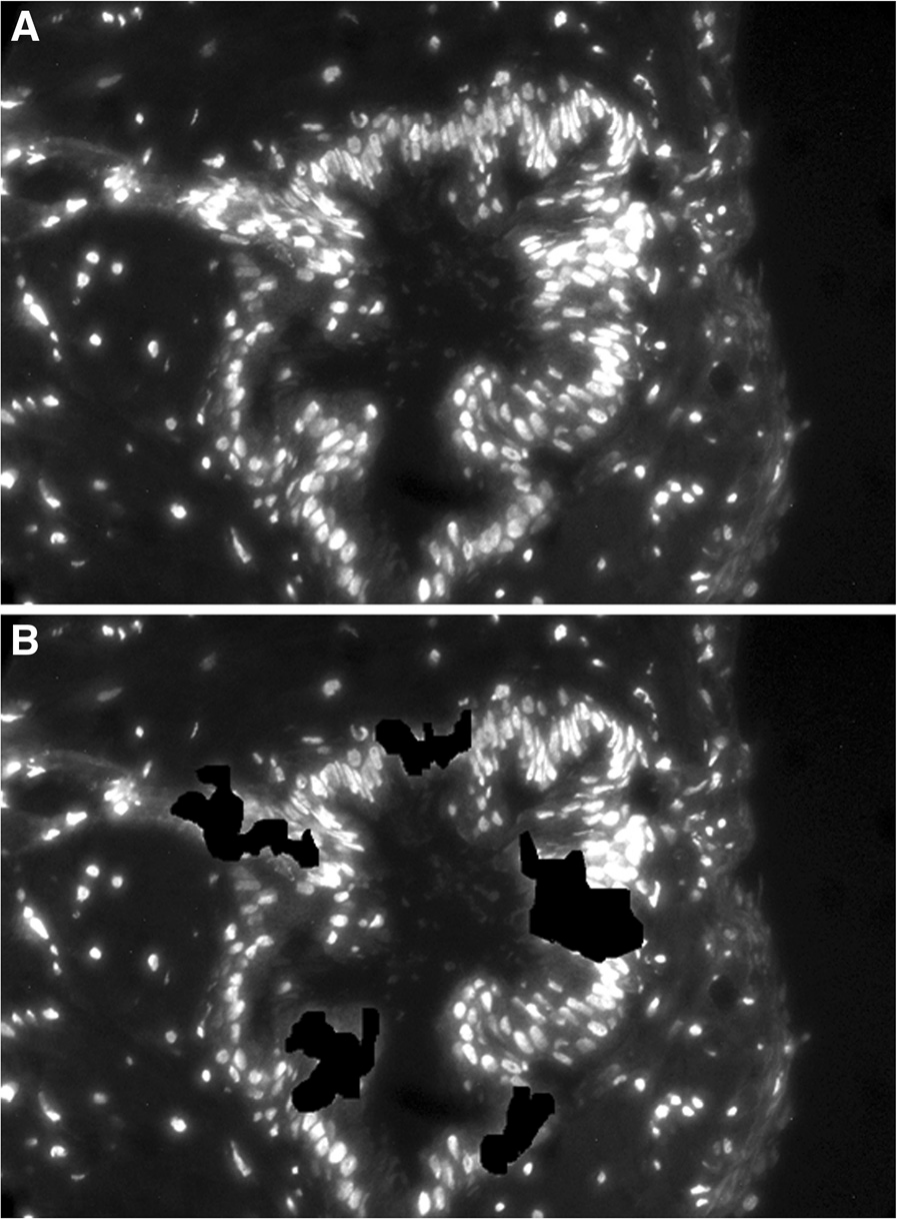
**Experiment 2: robustness of the algorithm.** Image **B** has been obtained from **A** by setting the intensity of random areas to null. This simulates an image with lower antigen expression.

In Experiment 2, 103 out of 106 (97.2%) (Table 3) were aligned correctly as shown in Figure 2. In order to simulate a situation where the antigen of interest (AR in this case) is present only in a limited area, we performed a test where we set the intensity of some random areas of the TRF image to null (Figure 4). Successful alignment was still obtained, however with less keypoints (data not shown).

**Table 3:** Experiments 2

	**H&E kyp**	**TRF kyp**	**Initial matches**	**(Best inliers)/(initial matches) (%)**	**Success rate % (#correct/#tot)**
Exp 2	9193	672	30	93%	97.2% (103/106)

Keypoints (kyp), initial matches, best inliers and success rate in experiment 2.

### Experiment 3

In Experiment 3, we performed registration between images of tissue sections, progressively further away from the respective initial section. As explained above, H&E stained sections were used.

Table 4 shows the results at distance *i* (1<*i*<3) from each other. The average number of initial matches and best inliers are calculated for comparison with progressively further sections. The average initial matches drop from 40.9 comparing consecutive sections, to around 10.4 comparing the furthest ones. Best inliers drop from 25.8 to around 3.4. For consecutive sections 93.9% of image pairs were correctly aligned, while this percentage dropped dramatically to 52.1% and 22% for the sections progressively further away.

**Table 4:** Experiment 3

**Distance from the reference section**	**Initial matches**	**Best inliers**	**Success rate %**
	**average (range)**	**average (range)**	**(#correct/#tot)**
consecutive + 1	40.9 (6–124)	25.8 (0–108)	93.9% (46/49)
consecutive + 2	13.5 (1–33)	5.6 (0–15)	52.1% (25/48)
consecutive + 3	10.4 (0–26)	3.4 (0–12)	22% (11/50)

Initial matches, best inliers and success rate for sections progressively further away from each other.

## Discussion

The present study shows that an automated registration of multimodal images from histological prostate sections is possible by using SIFT. Our work is novel with regards to multiple aspects: to our knowledge it is the first time that such algorithm is applied to multimodal microscopy for PCa. Our protocol can detect the presence of multiple antigens.

Pathologists often rely on integration of multiple immunohistochemical stainings (H&E, p63, and AMACR) to perform diagnostics. Experiment 1 shows that in at least 96.6% (visual evaluation) of the cases, the two differently stained sections can be automatically aligned. We therefore present a potential supportive tool for PCa diagnostics since this allows for automated alignment and fast visualization of areas of interest on differently stained sections. In addition, the automated approach displays advantages also for researchers with regards to time efficiency and management of large sample cohorts.

The quantitative evaluation in experiment 1, classified 82 out of 88 (93.2%) image pairs as correctly registered. Thus, the quantitative criterion rejected three image pairs that were originally accepted as correctly aligned by visual evaluation. The two evaluations agree in 96.5% of all cases. Of note, two of these three images failed to meet the quantitative criterion by a margin as small as one fifth of a pixel. If we accept also these images pairs as correctly aligned, then the agreement goes up to 98.8%. Based on these results we considered visual evaluation as a reliable way of assessing automated registration in all experiments.

In our protocol we have not only used different immunohistochemical staining but have also made use of TRF. TRF allows for biomarker quantification due to its signal linearity. As a result, we are able to integrate morphological information from H&E with quantitative analysis of the expression of a certain molecule (in this case AR). Biological studies on the AR status in prostate tissues using our method are ongoing. In the TRF and H&E registration we observed a success rate of 97% (103 out of 106 images). In addition, the performance of the algorithm seems to be very stable as displayed in the observation that 93% of the initial matches were identified as best inliers. Robustness to obstruction was also confirmed through correct alignment of corrupted images (Figure 4). However, the fact that we re-stained the same tissue section following removal of the antibody may have contributed to the good performance. In order to do the re-staining, we performed an optimization of the protocol. Nevertheless, these data show that reusing the same tissue again for re-staining does not affect the quality of the resulting images and the registration process.

In our third experiment we tested automated alignment of several consecutive H&E tissue section (4 in our initial setting). In our mind this would have given us the upper limit of sections that could be aligned and analysed for the expression of several biomarkers in the same area of interest. We observed that sections further than one section away from each other have already some substantial differences in the structure (assessed by an independent observer), which can explain the lower success rate in automated alignment. With this in consideration, the use of more than 3 consecutive sections for multiple staining analysis will not guarantee overlap of the area of interest. In order to address this issue, one could consider optimizing protocols for multilabeling of the same tissue section. In this regards, immunofluorescence would technically be the best solution for quantification.

Since PCa is a very heterogeneous disease we have assessed the performance of the algorithm for samples with different Gleason scores. The algorithm correctly aligned all the images with Gleason equal to or lower than 7, which is the most common Gleason score detected in patients. For patients with higher Gleason scores, 10 out of 13 images were correctly aligned. This may be due to the fact that the higher Gleason scores are characterized by a more complex structure. They present with fused glands and scattered individual cancer cells resulting in a highly variable appearance across consecutive sections.

Due to the fact that the image pairs in question have different modalities and may therefore not be easily aligned by minimizing an intensity-based dissimilarity measure, we have chosen a landmark-based registration method for the image alignment. An intensity-based dissimilarity measure was used in Kwak et al. [10] for images of different modalities; however this required first a transformation into binary images. In our method we use grayscale images in order to retain more of the available image information. Moreover, landmark-based methods focus on features two images have in common and ignore dissimilarities. In addition, landmark-based registration is also easier to compute and therefore a potentially faster method.

In our work the landmarks were extracted from the images by first detecting keypoints and descriptors using SIFT. True correspondences between keypoints were subsequently established using the descriptors and RANSAC. Both SIFT and RANSAC are well-established tools in computer vision and image analysis. The proposed method has been used for other medical registration problems, but the application to PCa and to these specific modalities (whose advantages have been explained before) is, to our knowledge, novel.

The time it takes to transform one image was used as a unit to measure the computational performance of the proposed algorithm. This is an operation fundamental to all registration problems and therefore appropriate for comparisons. We observed that 6% of the total time it takes to register two images (typically 1000×1000 pixels each) is used for the image transformation. In addition when analysing the performance in detail we found that the bottleneck of the algorithm is the computation of distance matrix, which is used to compare the keypoint descriptors derived from SIFT. This computation represents 40% of the total time required by the algorithm, which is nearly seven times the amount required for one image transformation. There are no algorithms able to find the exact nearest neighbor in a more efficient way than exhaustive search, however the Best Bin First [26] can speed up the computation by finding it with a certain probability. The average runtime of our script was 7.6 seconds per image pair, including visualizations, using a Matlab implementation. We have observed that a preprocessing step, which deletes all the spurious background keypoints, can reduce the distance matrix to 50% of its original size. Unfortunately, we have not been able to obtain information about the performance of the registration method described by Kwak et al. [10] However, we can infer from the method that they use that computation of the intensity-based dissimilarity measure requires one image transformation. Their optimization method (in four-dimensional space) requires five such computations just to get started and a number of iterations in order to converge to a good solution. Unless their method converges in about ten iterations, it cannot possibly be faster than the one proposed by us.

One must however mention that the current study may have some limitations. The work is a proof of principle study and therefore is performed on a limited number of samples. In addition, the samples come from one single institution and therefore the results must be validated by further studies conducted at several independent institutions.

## Conclusions

In this study we have investigated the potential to automatically align microscopic images of prostate tissue sections stained with different modalities. This addresses the need for integration of morphological information with protein expression data allowing for a more detailed description of PCa. Our results, based on the use of SIFT algorithm shows that potentially 3 consecutive sections of prostate tissue with different stainings can be aligned in an unsupervised way allowing for successive analysis of the tissue. Of note, good results were obtained when aligning H&E and p63/AMACR images (96.6% of images correctly aligned using visual evaluation) and even better results were obtained when aligning TRF and H&E images (97%). This shows that the algorithm performed well also with less informative images such as 1-channel TRF (it must be said that in this case using the same section for producing the 2 images might have contributed to the very high success rate). The advantage in terms of time efficiency is very clear when considering that typical research studies can include thousands of tissue samples and therefore thousands of comparisons that otherwise must be performed manually. The results in experiment 3 confirm what was observed in the other experiments and suggest that the number of easily alignable consecutive sections may be limited to 3. Therefore, if one wishes to investigate many biomarkers, it is preferable to develop multi-staining procedures to be performed on the same slide. Currently work in the field of clinically relevant image analysis remains limited. Our study is therefore a novel approach that supports implementation of automated image analysis in the field of PCa diagnostics and prognostics.

## Abbreviations

AMACR: Alpha-methylacyl-CoA racemase; H&E: Hematoxylin and eosin; TRF: Time resolved fluorescence; SIFT: Scale invariant feature transform; PCa: Prostate cancer; TRUS: Transrectal ultrasonography; RP: Radical prostatectomy; TMA: Tissue microarray; HGPIN: High grade prostate intraepithelial neoplasia; AR: Androgen receptor.

## Competing interests

The authors declare that they have no competing interests.

## Authors’ contributions

Design of the study: GL, AB, NCO. Collection of data: GL, NCO. Analysis and interpretation of data: GL, AB, NCO, AE, LH. Writing of the manuscript: GL, AB, NCO, AE, LH. Decision to submit the manuscript for publication: GL, AB, NCO, AE, LH. All authors read and approved the final manuscript.

## Pre-publication history

The pre-publication history for this paper can be accessed here:

http://www.biomedcentral.com/1471-2407/13/408/prepub
